# Smoking and mental health - impacts and implementation opportunities

**DOI:** 10.1192/j.eurpsy.2025.187

**Published:** 2025-08-26

**Authors:** J. Campion

**Affiliations:** SOUTH LONDON AND MAUDSLEY NHS FOUNDATION TRUST, London, United Kingdom

## Abstract

**Abstract:**

Jonathan will present the impacts of smoking on physical and mental health, the impact of smoking cessation on mental health and the need to reduce doses of some psychotropic medications after cessation in order to prevent toxicity. He will set out the low population coverage of evidence-based interventions for smoking cessation and prevention uptake particularly for people with mental health conditions, and the reasons for this. He will then outline implementable opportunities to scale up coordinated coverage of interventions by different sectors

**Disclosure of Interest:**

None Declared

